# Draft genome sequence of *Streptomyces* sp. strain G6 reveals biosynthetic gene clusters, including lanthipeptides with potential biomedical applications

**DOI:** 10.1128/mra.00815-25

**Published:** 2025-10-07

**Authors:** Michael Angelou L. Nada, Ursela G. Bigol

**Affiliations:** 1 Department of Science and Technology, Industrial Technology Development Institute 340935, Taguig, Philippines; Indiana University Bloomington, Bloomington, Indiana, USA

**Keywords:** Streptomyces, multidrug-resistant bacteria, draft genome, biosynthetic gene clusters, lanthipeptides, genome mining

## Abstract

The 7.20 Mbp draft genome of *Streptomyces* sp. strain G6 encodes multiple biosynthetic gene clusters, including posttranslationally modified peptides, nonribosomal peptides, and polyketide synthases. Genomic analysis suggests that G6 is a novel species, highlighting its potential for novel antibiotic discovery.

## ANNOUNCEMENT

*Streptomyces* is a genus of Gram-positive bacteria with genome sizes ranging from 4.8 to 13.6 Mbp (GC content: ~72%). They are known to synthesize a diverse array of metabolites with significant medicinal and biotechnological applications, resulting in the deposition of 3,569 *Streptomyces* genomes in NCBI RefSeq database (1). Seven *Streptomyces* strains have been isolated in the Philippines (2–5) and have demonstrated antibacterial activity against multidrug-resistant (MDR) bacteria (6), attributed to their production of bioactive compounds, such as chlorinated carbazole alkaloids (7). *Streptomyces* sp. strain G6 exhibits antagonistic activity against MDR clinical isolates of *Enterococcus faecalis* and *Acinetobacter baumannii* (DOST Annual Report). Here, we report its draft genome to support the discovery of bioactive secondary metabolites.

G6 was isolated from a soil sample collected at the Department of Science and Technology Garden, Taguig City, Philippines (14°29′22.1″N, 121°03′02.3″E) using serial dilution and plated on Tryptic Soy agar (Merck) plates for 5–7 days. Single colonies were propagated into 250 mL tryptic soy broth (TSB) and incubated at 30°C with shaking (175  rpm) for 4 days, forming dense yellow-orange bacterial pellet. Then, 1 mL culture was centrifuged (12,000 rpm, 15 min) to pellet bacterial cells. DNA was extracted using Genomic DNA Purification Kit (Promega #A1120). Sequencing library (15 pM) was prepared using Illumina DNA prep kit and sequenced on Illumina MiSeq platform (Illumina MiSeq Reagent Kit v3; 2 × 301 bp paired-end chemistry). Read quality was assessed using FastQC v0.11.9 (8) and trimmed with Trimmomatic v0.39 (Q-score:≥28; MinLen:100) (9), resulting in 3,110,139 raw sequences. Reads were assembled *de novo* using SPAdes v4.2.0 (--isolate) (10) and scaffolded using RagTag v2.1.0 (11). The reference strain *Streptomyces mutabilis* (GCF_014649815.1) was identified using TYGS platform (12). Genome quality, completeness, and coverage were determined using Quast v5.3.0 (13), CheckM2 v1.0.2 (14), BUSCO v5.8.0 (actinobacteria_phylum_odb10) (15), and BBMap v39.28 (16). The draft genome was annotated using the NCBI Prokaryotic Genome Annotation Pipeline v6.10 (17). Default parameters were used unless otherwise stated.

**Fig 1:**
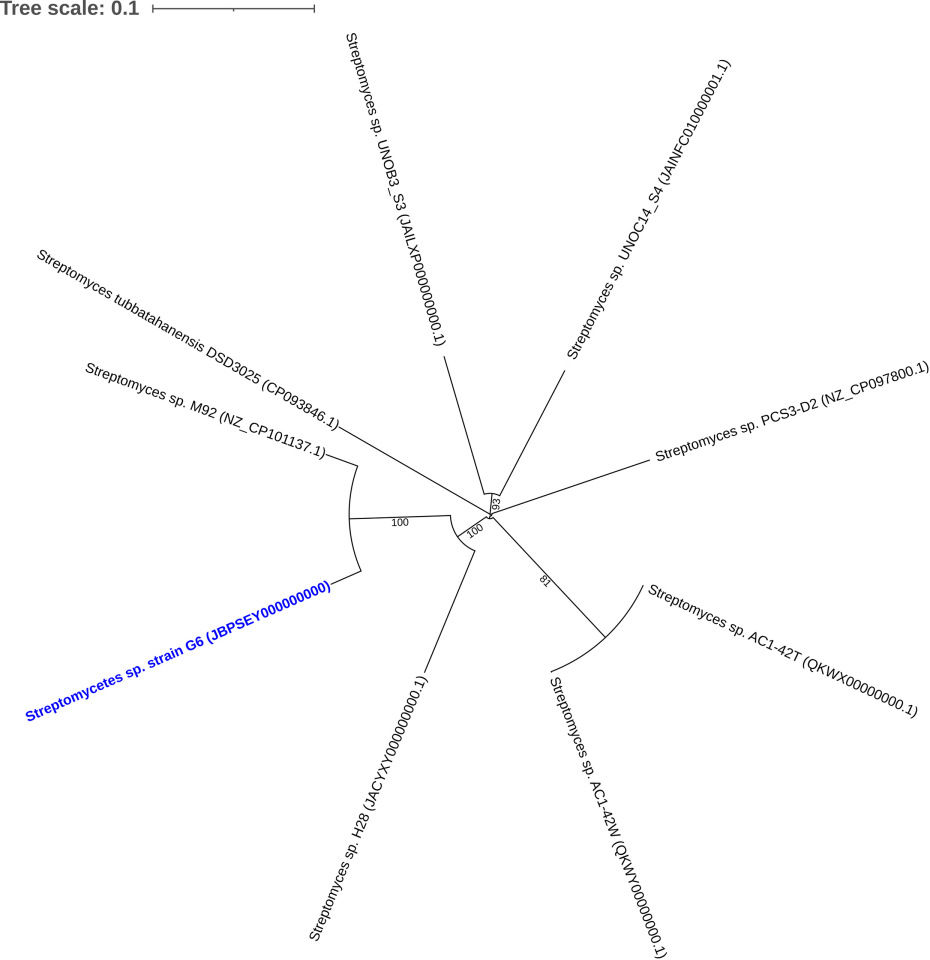
Phylogenomic tree of *Streptomyces* sp. strain G6 and other isolates from the Philippines. Branch numbers indicate bootstrap support values (100 replicates), with an average branch support of 80.0%. The branch lengths were scaled in terms of Genome BLAST Distance Phylogeny (GBDP) distance formula *d_5_* . The tree was rooted at midpoint. Pairwise genome comparison between *Streptomyces* sp. G6 and *Streptomyces* sp. M92 show 96.2% ANI and 65.5% dDDH, suggesting close but potentially distinct species-level relatedness. Full pairwise genome comparisons between *Streptomyces* sp. strain G6 and other isolates are available on Figshare. The S*treptomyces*-specific delineation thresholds are ≥96.7% ANI and ≥70 dDDH.

G6 has a genome size of 7.20 Mbp (GC content: 71.8%; coverage: 162×). The draft genome (CheckM2 completion: 99.9%, contamination: 0.42%; BUSCO: 93.8%), composed of 29 contigs (N50 >7,173 Kbp), has 6,982 coding sequences (coding density: 88.4%) and 67 tRNAs. Three plasmids and one prophage region were detected using geNomad v1.11.1 (18). Taxonomic classification was performed using GTDB-Tk v2.4.1 and the GTDB database v220 (19, 20), which identified *Streptomyces* sp. M92 (GCF_028473745.1) as the closest relative (96.1% ANI). The dDDH value (65.5%), calculated using Genome-To-Genome Distance Calculator v3.0 (12), suggests that G6 is a distinct species closely related to M92 (Fig. 1), based on *Streptomyces*-specific delineation thresholds (21).

AntiSMASH v8.0 (22) and BAGEL4 (23) identified 25 biosynthetic gene cluster (BGC) regions (Table 1), including those involved in the production of terpenes and their precursor, nonribosomal peptides (NRPS), polyketide synthase (Type I, II, III PKS), siderophores, indole, ectoine, and other metabolites commonly observed in *Streptomyces* isolates. Notably, clusters involved in the production of ribosomally synthesized and post-translationally modified peptides (RiPPs), such as lanthipeptides and planosporicin, were also detected. These small peptides are known for their antimicrobial and antiviral activities (24). Future validation of these bioactive compounds may be undertaken to confirm their biological activity.

**TABLE 1:** Biosynthetic gene clusters in *Streptomyces* sp. strain G6 predicted by AntiSMASH v8.0.1^*a*^

Region	Type	Location in the genome	Most similar known cluster	Similarity confidence
Region 1.1	Ectoine	76,330-86,728	Ectoine	High
Region 1.2	Melanin	969,821–980,435	–	–
Region 1.3	NI-siderophore	1,056,379–1,086,151	Desferrioxamin B/desferrioxamine E	High
Region 1.4	NRPS	1,239,461–1,301,423	Sarpeptin A / sarpeptin B	Low
Region 1.5	Lanthipeptide class V	1,859,177–1,905,374	Pristinin A3	Low
Region 1.6	Lanthipeptide class I	2,167,140–2,191,821	Planosporicin	High
Region 1.7	Terpene	2,984,468–3,005,562	Albaflavenone	High
Region 1.8	Terpene-precursor	3,014,127–3,035,263	–	–
Region 1.9	T2PKS	3,045,612–3,118,157	Spore pigment	Medium
Region 1.10	NI-siderophore	3,542,356–3,572,287	Kinamycin	Low
Region 1.11	NRP-metallophore, NRPS	3,632,177–3,695,619	Coelibactin	100%
Region 1.12	RiPP-like	3,795,770–3,807,107	–	–
Region 1.13	Terpene	3,823,888–3,846,068	Geosmin	High
Region 1.14	NI-siderophore	3,981,331–4,012,496	–	
Region 1.15	T2PKS, Lanthipeptide class I	4,255,863–4,328,354	Rubiginone A2/rubiginone J/rubiginone K/rubiginone L/rubiginone M/rubiginone N/ochromycinone/rubiginone B2	High
Region 1.16	Terpene	4,689,446–4,716,197	Hopene	High
Region 1.17	Hydrogen-cyanide	4,832,899–4,845,784	–	–
Region 1.18	Lanthipeptide class I	4,952,205–4,978,542	–	–
Region 1.19	NRPS, T1PKS, butyrolactone	5,064,116–5,178,515	C-1027	Low
Region 1.20	Terpene, T1PKS,	5,260,095–5,315,767	2-Methylisoborneol	High
Region 1.21	Indole	5,507,691–5,528,818	5-Dimethylallylindole-3-acetonitrile	High
Region 1.22	Terpene	5,587,287–5,626,147	Isorenieratene	Medium
Region 1.23	T3PKS	6,048,991–6,090,082	Flaviolin/1,3,6,8-tetrahydroxynaphthalene	High
Region 3.1	Lanthipeptide class I, T3PKS,	35,819–91,698	2-Methoxy-5-methyl-6-(13-methyltetradecyl)−1,4-benzoquinone/2-methoxy-5-methyl-6-(13-methyltetradecyl)phenol	High
Region 3.2	Terpene precursor	147,273–168,415	Isorenieraten	Low

^*a*^ “–” indicates information is not available.

## Data Availability

The raw reads and genome sequence of *Streptomyces sp*. strain G6 were deposited in NCBI Sequence Read Archive (SRA) and DDBJ/ENA/GenBank database under Bioproject PRJNA1288564 and accession number JBPSEY000000000, respectively. The version described in this paper is version JBPSEY010000000.
